# Cocreating a programme to prevent injuries and improve performance in Australian Police Force recruits: consumer, industry partner and researcher involvement protocol

**DOI:** 10.1136/bmjsem-2025-002632

**Published:** 2025-05-26

**Authors:** Myles Calder Murphy, Andrea Bruder, Vanessa R Sutton, Garth Allen, Andrea Mosler, Jonathan Hodgson, Simone Radavelli-Bagatini, Evert Verhagen, Joanne Kemp, Caroline Jones, Joletta Belton, Debra Langridge

**Affiliations:** 1 Nutrition and Health Innovation Research Institute, School of Medical and Health Sciences, Edith Cowan University, Joondalup, Western Australia, Australia; 2 Institute for Health Research, The University of Notre Dame Australia, Fremantle, Western Australia, Australia; 3 La Trobe Sport and Exercise Medicine Research Centre, La Trobe University, Melbourne, Victoria, Australia; 4 Discipline of Physiotherapy, Podiatry, Prosthetics and Orthotics, School of Allied Health, Human Services and Sport, La Trobe University, Melbourne, Victoria, Australia; 5 Consumer and Community (Patient and Public) Partner, Joondalup, Western Australia, Australia; 6 Physical Training Unit, Western Australia Police Academy, Joondalup, Western Australia, Australia; 7 Australian IOC Research Centre, La Trobe University, Melbourne, Victoria, Australia; 8 Amsterdam Collaboration on Health & Safety in Sports, Department of Public and Occupational Health, Amsterdam Movement Sciences, Vrije Universiteit Amsterdam, Amsterdam, Netherlands; 9 Union of European Football Associations, Nyon, Switzerland; 10 Consumer and Community Involvement Program (CCIProgram), Western Australian Health Translation Network, The University of Western Australia, Perth, Western Australia, Australia; 11 Consumer and Community (Patient and Public) Partner, Colorado, Colorado, USA

**Keywords:** Injuries, Prevention, Qualitative

## Abstract

We are conducting a research program to cocreate, implement and evaluate an injury prevention intervention for the Western Australia (WA) Police Force Recruit Training Academy. This programme of research has three primary phases: (1) cocreate an injury prevention intervention for the WA Police Force with WA Force recruits, WA Police Force staff, health professionals and injury prevention experts, (2) implement the injury prevention intervention into WA Police Force Recruit training and (3) evaluate the reach, effectiveness, adoption, implementation and maintenance of the injury prevention intervention. Our research programme includes the involvement of consumers, industry partners and researchers. To ensure collaboration and to measure our consumer, industry partner and researcher involvement, we have developed a protocol, including qualitative and quantitative evaluation, to address potential barriers to involvement. Thus, this protocol details our consumer, industry partner and researcher involvement plan across all three phases of this 5-year project and how we will evaluate their experience and influence. Our primary objective is to ensure meaningful consumer, industry partner and researcher involvement at all stages of the research process and evaluate how the research programme was influenced by consumer, industry partner and researcher involvement.

**WHAT IS ALREADY KNOWN ON THIS TOPIC**

- The involvement of consumer and industry partners benefit health research and is likely to improve research translation.

**WHAT THIS STUDY ADDS**

- This protocol details our consumer, industry and research partner involvement plan and how this involvement will be supported and monitored.

**HOW THIS STUDY MIGHT AFFECT RESEARCH, PRACTICE OR POLICY**

- By involving consumer, industry and research partners throughout our research, we hope to improve the outcomes of our 5-year research programme.

## Background

 Police Force recruits have a substantial injury burden due to the high prevalence, incidence and severity of musculoskeletal injury during their recruit training.^1^,^3^ While a substantial number of risk factors for injury occurrence have been investigated,^4^ and general recruit training programmes have been described,^5 6^ there is a dearth of research into prevention interventions.

We aim to address this research and practice gap. We are conducting a research programme to cocreate, implement and evaluate an injury prevention intervention for the Western Australia (WA) Police Force Recruit Training Academy. This programme of research has three primary phases:

1. Cocreate an injury prevention intervention for the WA Police Force with WA Force recruits, WA Police Force staff, health professionals and injury prevention experts.
2. Implement the injury prevention intervention into WA Police Force Recruit training.
3. Evaluate the reach, effectiveness, adoption, implementation and maintenance of the injury prevention intervention.

Our research programme includes the involvement of consumers, industry partners and researchers. For this project, ‘consumers’ refers to people injured during tactical training and current tactical officers (eg, Police Force officers). The term ‘industry partner’ refers to staff within the Police Force who are involved in training delivery and injury prevention (eg, physical trainers or executive staff).

It has long been established that involving consumers (also known as patient and public partners) when developing a research programme leads to increased trust^7^ as well as enhanced healthcare outcomes and impact.^8 9^ Likewise, the involvement of other key partners (eg, industry) is encouraged for successful research translation and implementation.^10 11^ Consumer involvement in sport and exercise medicine research is a priority,^12^ and templates for consumer involvement in musculoskeletal research currently exist.^13^ However, several barriers to integrating consumer involvement in health research have been identified.^14^ Specifically, the following generic barriers to consumer involvement in research have been identified: (1) lack of connection with researchers/research projects, (2) low research literacy, (3) structural barriers, (4) lack of acknowledgement, (5) implementation challenges, (6) inadequate information provision and (7) representation concerns.^14^

To ensure collaboration and to measure our consumer, industry partner and researcher involvement, we have developed a protocol, including qualitative and quantitative evaluation, to address potential barriers to involvement. Thus, this protocol details our consumer, industry partner and researcher involvement plan across all three phases of this 5-year project and how we will evaluate their experience and influence.

### Research questions

1. Do we have meaningful consumer, industry partner and researcher involvement, as reported by consumers, industry partners and researchers?
2. How does consumer, industry partner and researcher involvement influence the research programme?

### Objective

We will implement the consumer, industry partner and researcher involvement plan described in this protocol. Our primary objective is to ensure meaningful consumer, industry partner and researcher involvement at all stages of the research process and evaluate how the research programme was influenced by consumer, industry partner and researcher involvement. Thus, our specific objectives are

1. Evaluate the level of consumer, industry partner and researcher involvement in this research programme, as reported by consumers, industry partners and researchers.
2. Explore how consumer, industry partner and researcher involvement influenced this research programme.

## Methods

Phase 1 of the research project that this protocol relates to has been published in detail elsewhere.^15^

### Reporting guideline

This protocol has been guided by the Australian National Health and Medical Research Centre Statement on Consumer and Community Involvement in Health and Medical Research (https://www.nhmrc.gov.au/about-us/consumer-and-community-involvement/consumer-and-community-engagement) and the Guidance for Reporting Involvement of Patients and the Public (GRIPP2) reporting checklists, which were designed to improve reporting of patient and public involvement in research.^16 17^

### Project governance

Our research project has an overall project steering committee that consists of seven project leads: (1) Dr Myles Murphy, (2) Professor Sophia Nimphius, (3) Dr Andrea Mosler, (4) Dr Andrea Bruder, (5) chairperson of the consumer advisory group, (6) chairperson of the industry partner advisory group and (7) chairperson of the research advisory group.

### Advisory group structure

**Figure 1:**
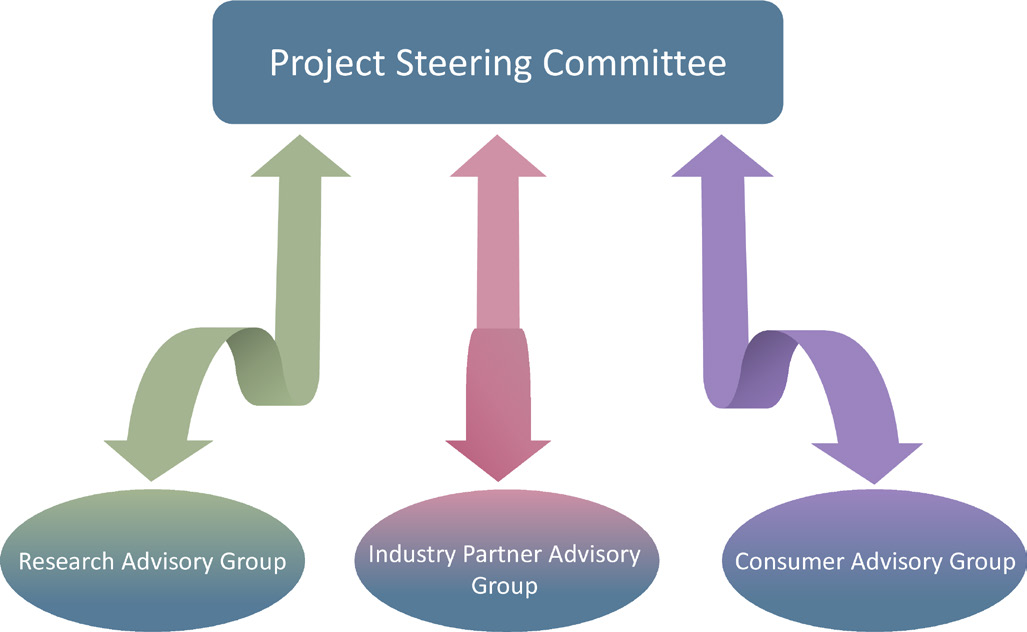
Research governance structure.

Our established governance structure, which was developed before our funding submission, includes a project steering committee with executive decision-making for all aspects of the research project. Our governance structure also includes one consumer advisory group, one industry partner advisory group and one research advisory group to collectively inform the decisions of the project steering committee. Each of the three advisory groups will consist of at least five people, thus positioning them to offer diverse opinions to the steering committee on all aspects of the project (figure 1). Consumer and industry advisory group members have already been identified via purposive sampling to ensure diverse demographic characteristics and various job roles. For the consumer advisory group, appropriate consumers were identified through the steering committee’s networks and were nominated for inclusion (noting consumers did not have to accept nomination). The key industry collaborators were identified and invited to participate in the industry advisory group (noting industry partners did not have to accept nominations). The research advisory group was established in the development of the funding application. Thus, all advisory group members have been identified and indicated an ability to commence as members in 2025.

The consumer advisory group will be chaired by a member of the advisory group who has lived experience of injury from tactical training. Mr Garth Allen, the manager of the physical training unit for the WA Police Force, will chair the industry partner advisory group. Both consumer and industry partner advisory groups will also have a deputy chairperson, Caroline Jones, a trained instructor for the Western Australia Health Translation Network (WAHTN) Consumer and Community Involvement Program (CCIProgram) for the first year. During this year, Ms Vanessa Sutton will shadow Caroline Jones throughout this project, so she is positioned to take over this role as deputy chairperson on both the consumer and industry partner advisory groups in years 2–5. A postdoctoral research fellow employed through this grant project will be the secretary for all three advisory groups.

The chairperson for each of the three advisory groups will also act as a representative on the project steering committee, ensuring all communications and deliverables from advisory groups are directly conveyed to the decision-making body (the project steering committee). Consumer, industry partner and research advisory groups will meet regularly throughout the project, with the meeting schedule outlined in table 1. The consumer, industry partner and research advisory groups were split due to the potential perceived unequal relationships between WA Police Force recruits and WA Police Force staff, reducing the impact of power imbalances in our advisory groups.^18^

**Table 1:** Consumer and industry partner advisory groups will meet following the schedule below, where shaded areas indicate a planned meeting

Phase	Year	Group	Quarter 1 (January to March)	Quarter 2 (April to June)	Quarter 3 (July to September)	Quarter 4 (October to December)
One	2025	Consumer				
		Industry				
		Research				
Two	2026	Consumer				
		Industry				
		Research				
	2027	Consumer				
		Industry				
		Research				
	2028	Consumer				
		Industry				
		Research				
Three	2029	Consumer				
		Industry				
		Research				

#### Eligibility and representation

While our provisional advisory groups have been selected, we appreciate they are likely to change over the 5-year project and have detailed the requirements for current and future members. To be eligible for inclusion in the consumer advisory group, members must be adults over 18 years with lived experience of working in the WA Police Force in the previous 12 months and/or having had an injury during tactical training, resulting in an inability or impaired ability to work. As we aim to retain our consumers for the 5-year project duration, and recruit training is only 6 months long, our consumers are not restricted to current WA Police Force recruits.

To be eligible for inclusion in the industry partner advisory group, members must be WA Police Force staff involved in administering and delivering WA Police Force recruit training (eg, physical trainers, police instructors, WA Police Force health staff, WA Police Force executive staff).

The chief and associate investigators with an academic affiliation from the funding application were included in the research advisory group and are listed elsewhere.^15^ Diversity within advisory groups is encouraged, and we followed diversity and inclusion recommendations to inform group member selection. Specifically, where able, we considered a balance of age, gender identity, geographical location, academic and professional affiliations, career stage sector, profession and level of education. Members of both our consumer and industry partner advisory groups will be remunerated as per the WAHTN CCIProgram Honorarium Guidelines (https://cciprogram.org/wp-content/uploads/sites/2/2024/09/CCIP-Honorarium-Guidelines-September-2024.pdf) as their roles on advisory groups do not fall within their workloads. The consumer and industry partner advisory group chairperson will be remunerated at $A75.00 per hour. General consumer and industry partner advisory group members will be remunerated at $A37.50 AUD per hour.

### Training

#### Consumers and industry partners

Consumer and industry advisory group members will receive training via the WAHTN CCIProgram (https://cciprogram.org/events-and-training/). This 1-hour training programme will cover aspects of the foundations of involvement of the lived experience and ‘stakeholder’ voice in research, Advisory Group governance, developing and confirming the Group’s Terms of Reference and Advisory Group conduct.

#### Research team

All members of the research advisory group will complete the WAHTN CCIProgram’s Introduction to CCI in Health Research and the Masterclass online eCourse (https://cciprogram.org/), or an equivalent program, which includes topics such as how to communicate research to non-academic collaborators.

### Participation

Following the International Association Public Participation Spectrum,^19^ our consumer, industry and research advisory groups will collaborate with the steering committee to ensure they are involved in all aspects of the decision-making process; specific involvement is described below.

#### Preparation

Our funding application, preliminary methodology, governance structure and protocol were codesigned with individual consumers, industry partners and the WAHTN CCIProgram.

#### Phase 1 (intervention development)

The specific details of how our consumer and industry partner advisory groups will be integrated into phase 1 have been described.^15^ Our consumer, industry partner and research advisory groups will review and interpret the results of the concept mapping and focus groups provided to them by the steering committee (with an effort made to ensure the results are plain language). Finally, all advisory groups will provide input into the final intervention. Quarterly meetings will be performed during phase 1 (year 1), with an agenda, including a research update, provided 2–4 weeks before each meeting.

#### Phase 2 (intervention implementation)

Our consumer, industry partner and research advisory groups will inform how we collect data for the evaluation of the injury prevention intervention’s reach, adoption, implementation and maintenance.^20 21^ For example, each group will make recommendations on how best to evaluate the reach of the injury prevention intervention, and who should be included in the evaluation of reach, with the final approach decided by the steering committee.

Data on effectiveness (injury burden; retention rate; economic costs) will be routinely collected. Once the intervention has been implemented (estimated in the second quarter of 2026), advisory group meetings will be less frequent (two times per year) as the project will be in a maintenance phase until the end of the implementation period (fourth quarter of 2028). Meetings will be preceded by an agenda, including a research update, provided 2–4 weeks before the meeting. To ensure ongoing/continued engagement and awareness, additional research updates will also be communicated to all advisory group members in the quarters when there is no scheduled meeting.

#### Phase 3 (intervention evaluation)

Our consumer, industry partner and research advisory groups will review and interpret the results of our analyses of the injury prevention intervention reach, effectiveness (injury burden; retention rate; economic costs), adoption, implementation and maintenance. We anticipate preliminary results to be available in the second quarter of 2029, so our advisory groups will meet quarterly. An agenda and research update will be provided to members 2–4 weeks before the meeting.

### Acknowledgement

Due to their importance in the research project, consumers, industry partners and research advisory group members will be offered authorship or acknowledgement based on their preferences and contributions to the project in any outputs from this project. For all three advisory group members, authorship or acknowledgement of the outputs generated in phases 1, 2 and 3 will be based on the Contributor Roles Taxonomy (CRediT) statement.^22^

#### Reporting

The GRIPP2-Short Form will guide reporting in all publications relating to the primary research outcomes as a reporting checklist specifically designed for the reporting of patient and public involvement in research. The GRIPP2-Long Form will guide reporting in all publications relating to consumer, industry partner and researcher collaboration and/or partnership as a more extensive checklist with better alignment to our consumer, industry partner and researcher involvement.

### Dissemination

The steering committee may invite consumer and industry partner group members to present research findings. This will assist in disseminating our research findings to networks beyond those traditionally used by researchers. Our consumer and industry partner advisory group members will be remunerated as per the WAHTN CCIProgram Honorarium Guidelines when delivering our research presentation. Thus, members will be remunerated at $A148.00 per presenter, per presentation.

## Evaluation

### Objective 1

Evaluate the level of consumer, industry partner and researcher involvement in this research program, as reported by consumers, industry partners and researchers.

We will evaluate the advisory group experiences similarly to methods we have used previously,^23^ to determine if we achieved ‘meaningful’ involvement, as rated by the advisory group members. However, differences from the methodology we have previously used will exist, given the differences in the purposes of our advisory groups (project delivery focus) compared to those of our prior research (exploratory focus).^23^

#### Advisory group structure and governance

The first meeting with consumer and industry partner advisory groups will focus on exploring member expectations and understanding the advisory group’s purpose. The facilitator and deputy chair, Caroline Jones, will develop group expectations, conditions of engagement and terms of reference for each advisory group as per current WAHTN CCIProgram practice and international recommendations.^24^

The structure and governance of the advisory groups will be reviewed every 6 months via an anonymous survey sent to all members of the consumer, industry partner and research advisory groups for feedback on key aspects of advisory group success. Specifically, based on processes from our existing advisory groups, we will ask the following questions: (1) in your own words, what is working well, (2) in your own words, what are the challenges and (3) in your own words, what could be improved.^23^ If feasible, feedback provided via these 6 monthly reviews will be addressed.

#### Advisory group members

All consumers, industry partners and research advisory group members will complete a formal, annual evaluation of their experiences with our partnership and collaboration strategy to evaluate current perceptions. All consumer, industry partner and research advisory group members will use the Public and Patient Engagement Evaluation Tool: Participant Questionnaire (Module B. Ongoing/Long-term Engagement Initiatives).^25^ In addition, consumers will reflect on their experiences using the 22-item self-reported Patient Engagement in Research Scale,^26^ codesigned with patient (consumer) partners.

The Public and Patient Engagement Evaluation Tool: The Patient Questionnaire is a 21-item outcome measure with a mixture of both a 5-point Likert scale and open-text responses.^25^ The Public and Patient Engagement Evaluation Tool evaluates four constructs: (1) communication and support for participation, (2) sharing your views and perspectives, (3) impacts and influence of the engagement initiative and (4) final thoughts.^25^

The Patient Engagement in Research Scale has acceptable content validity and measures eight constructs related to the research experience: (1) procedural requirements, (2) convenience, (3) contributions, (4) support, (5) team interaction, (6) research environment, (7) feel valued and (8) benefits.^26^ The Patient Engagement in Research Scale has acceptable internal consistency (ordinal alpha=0.96), acceptable reliability (intraclass correlation coefficient=0.86) and structural validity when evaluated via Rasch measurement theory.^27^

#### Industry partners

All industry partner and research advisory group members will complete a formal, annual evaluation of their organisational experiences of our partnership and collaboration strategy. The industry partner and researcher advisory groups experience will be evaluated via the Public and Patient Engagement Evaluation Tool: Organizational Questionnaire,^25^ and the Victorian Health Promotion Foundation Partnership Analysis Tool (https://www.vichealth.vic.gov.au/sites/default/files/VHP-part-toollow-res.pdf).

The Public and Patient Engagement Evaluation Tool: The Organisation Questionnaire is a 30-item outcome measure with a mixture of both a 5-point Likert scale and open-text responses.^25^ The Public and Patient Engagement Evaluation Tool evaluates four constructs: (1) policies and practices that support public and patient engagement, (2) participatory culture, (3) influence and impact, (4) collaboration and common purpose and (5) final thoughts.^25^ The Victorian Health Promotion Foundation Partnership Analysis Tool has been previously used in musculoskeletal injury prevention research.^28^ The Victorian Health Promotion Foundation Partnership Analysis Tool consists of seven domains: (1) determining the need for the partnership, (2) choosing partners, (3) making sure partnerships work, (4) planning collaborative action, (5) implementing collaborative action, (6) minimising the barriers to partnerships and (7) reflecting on and continuing the partnership. Each domain consists of five items scored on a 1–5 scale, with 1 indicating strong disagreement and 5 indicating strong agreement.

#### Analysis plan

We will describe the outcomes from our consumer, industry partner and research advisory group member outcome measures at all time points. Statistical analysis will be performed using repeated measures models to evaluate how our engagement changes over time.

### Objective 2

Explore how consumer, industry partner and researcher involvement influenced this research program.

As self-reported experiences (ie, those described in the evaluation of objective one) can be prone to bias given their subjective nature, we will also formally report what changes were made to our research programme as a direct result of our consumer, industry partner and researcher involvement. Thus, in addition to including the information required by the formal GRIPP2 checklists to improve transparency and highlight the contribution of our consumer, industry partner and research advisory groups, the recommendations made by the advisory groups and whether they were or were not actioned by the steering committee (ie, what parts of the project changed as a direct result of advisory group recommendations) will be compiled and formally reported (eg, publicly available report or journal publication).

## Significance

The protocol aims to enhance the meaningful involvement of our consumers, industry partners and research advisory groups. By establishing a governance structure involving consumers and industry partners, we aim to address the potential ‘lack of connection with researchers/research projects’. We aim to address the potential for ‘low research literacy’ by providing specific training to consumers, industry partners and researchers. By providing remuneration, we aim to address ‘structural barriers’ and recognise the expertise these members contribute to the processes and overall project. By following the CRediT statement and specifically reporting the changes made to the project based on the recommendations of the advisory groups, we aim to address the potential for a ‘lack of acknowledgement’. We aim to address ‘implementation challenges’ by including an industry partner advisory group and a consumer advisory group. By including advisory group chairpersons on the steering committee, performing regular communication via email or meeting and establishing group expectations and terms of reference, we aim to address the potential for ‘inadequate information provision’. Finally, by including a diverse sample within the consumer, industry partner and research advisory groups, we aim to address any potential ‘representation concerns’. The effectiveness of our involvement strategy will be evaluated, reported and addressed as possible.

## Data Availability

No data are available.
